# Downregulation of Pro-Inflammatory and Pro-Angiogenic Pathways in Prostate Cancer Cells by a Polyphenol-Rich Extract from Olive Mill Wastewater

**DOI:** 10.3390/ijms20020307

**Published:** 2019-01-14

**Authors:** Denisa Baci, Matteo Gallazzi, Caterina Cascini, Matilde Tramacere, Daniela De Stefano, Antonino Bruno, Douglas M. Noonan, Adriana Albini

**Affiliations:** 1 School of Medicine and Surgery, University of Milano-Bicocca, 20900 Monza, Italy; denisa.baci@multimedica.it (D.B.); albini.adriana@gmail.com (A.A.); 2 Scientific and Technology Pole, IRCCS MultiMedica, 20138 Milano, Italy; matteo.gallazzi@virgilio.it (M.G.); caterina.cascini91@gmail.com (C.C.); matildeelena.tramacere@gmail.com (M.T.); daniela.destefano@multimedica.it (D.D.S.); douglas.noonan@uninsubria.it (D.M.N.); 3 Department of Biotechnology and Life Sciences, Laboratory of Immunology and General Pathology, University of Insubria, 21100 Varese, Italy

**Keywords:** olive mill wastewater, polyphenols, hydroxytyrosol, prevention, prostate cancer

## Abstract

Dietary phytochemicals are particularly attractive for chemoprevention and are able to modulate several signal transduction pathways linked with cancer. Olive oil, a major component of the Mediterranean diet, is an abundant source of phenolic compounds. Olive oil production is associated with the generation of a waste material, termed ‘olive mill wastewater’ (OMWW) that have been reported to contain water-soluble polyphenols. Prostate cancer (PCa) is considered as an ideal cancer type for chemopreventive approaches, due to its wide incidence but relatively long latency period and progression time. Here, we investigated activities associated with potential preventive properties of a polyphenol-rich olive mill wastewater extract, OMWW (A009), on three in vitro models of PCa. A009 was able to inhibit PCa cell proliferation, adhesion, migration, and invasion. Molecularly, we found that A009 targeted NF-κB and reduced pro-angiogenic growth factor, VEGF, CXCL8, and CXCL12 production. IL-6/STAT3 axis was also regulated by the extract. A009 shows promising properties, and purified hydroxytyrosol (HyT), the major polyphenol component of A009, was also active but not always as effective as A009. Finally, our results support the idea of repositioning a food waste-derived material for nutraceutical employment, with environmental and industrial cost management benefits.

## 1. Introduction

Adherence to a Mediterranean dietary pattern has generally been associated with a decreased risk of developing most non-communicable diseases, including cardiovascular, neurodegenerative pathologies, and cancer [1,2,3,4]. This has led to active exploration of whole and isolated products from different naturally occurring sources in various preclinical and clinical settings [5,6,7,8,9]. Chemopreventive activities of diverse diet-derived phytochemicals have been widely studied in the most common cancers, including lung, colon, breast, and prostate. Chemopreventive agents are natural or synthetic agents that prevent or delay cancer development, progression, and/or recurrence [5,6,7], and many natural products and dietary agents have chemopreventive properties. These chemopreventive activities include anti-inflammatory and antioxidant properties [5,6,10,11,12,13].

Among other beneficial foods, olive oil is considered a reservoir of chemopreventive molecules [14]. Extra virgin olive oil (EVOO) is the fresh olive juice obtained exclusively by mechanical and physical processes of olive extraction. It consists of a major fraction of triacylglycerides representing more than 98% of the total weight, whereas the minor fraction (approximately 2% of the weight) is represented by of a complex set of compounds, which includes over 230 molecules. Different groups of phenolic compounds can be found in EVOO: phenolic acids (caffeic, vanillic, syringic, *p*-coumaric, *o*-coumaric, protocatechuic, synaptic, *p*-hydroxybenzoic, and gallic acid) and phenolic alcohols, principally represented by 3,4-dihydroxyphenylethanol (3,4-DHPEA, hydroxytyrosol) and (*p*-hydroxyphenyl)ethanol (*p*-HPEA). Current data strongly support the fact that the beneficial effects of olive oil consumption are also due to its minor bioactive components [14].

Large volumes of wastewater (olive mill wastewater; OMWW) are generated during olive oil production, particularly during the malaxation process (continuous washing of olive paste with warm water prior to the procedure of separation of the oil from the solid fraction [15,16]). OMWWs are rich in polyphenols associated with beneficial effects, that include the metabolic compartment [17]. We previously reported that a polyphenol-rich purified extract from OMWW, termed A009, which is particularly rich in hydroxytyrosol (HyT), determines anti-angiogenic effects both in endothelial cells in vitro and in an angiogenesis model in vivo [18]. We further demonstrated that A009 and HyT prevent human colon cancer cell proliferation in vitro and tumor growth in vivo [19]. Here, we investigated the effects of A009, and HyT alone, on human prostate cancer cell adhesion, proliferation, migration, and invasion, features of cancer development and progression.

Despite recent advances in diagnosis and treatment, prostate cancer (PCa) remains one of the leading causes of cancer-related deaths in men worldwide [20]. Currently available treatments can increase 5-year survival in the early stages of PCa, but the metastatic disease is still difficult to manage [21]. In addition, current diagnostic approaches are inadequate, in particular, prostate-specific antigen (PSA) is a prostate, but not cancer, specific biomarker, and repeated biopsies are required to distinguish between benign prostate hyperplasia (BPH) and PCa [22]. Epidemiological and clinical studies strongly support the association between nutrition and development or progression of major cancers [23], including PCa [24]. This complex scenario clearly suggests that strategies aimed at preventing PCa insurgence are urgently needed. As a consequence of its long latency period, PCa is considered as an ideal cancer type for chemopreventive approaches that also include dietary interventions.

Inflammatory responses are involved in cancer initiation and metastasis [25,26,27]. Chemotherapy and radiotherapy can, themselves, induce inflammation [28,29]. NF-κB activation and many other signals associated with inflammation are known to contribute to prostate cancer development [30,31,32,33,34]. The activation of NF-κB controls the expression of numerous proinflammatory genes, including interleukin 6 (IL-6), that is highly expressed in most prostate tumors and regulates key functions, such as proliferation, apoptosis, angiogenesis, and differentiation [35]. Several studies have shown that IL-6 is implicated in the regulation of cellular stemness by increasing phosphorylation of signal transducer and activator of transcription STAT3. IL-6/STAT3 signaling is a potential target of experimental therapies to improve PCa treatment.

Here, we focused on the properties of the A009 and the equivalent HyT concentration by investigating the capabilities to block PCa cell aggressiveness in vitro, and analyzed the cellular mechanisms involved, in a potential chemopreventive approach. We showed that the tested extracts inhibit cytokines involved in angiogenesis by reducing the release of pro-angiogenic factors: VEGF, CXCL8, and CXCL12. Furthermore, A009 blocked the pro-inflammatory pathways NF-κB and IL-6/STAT3.

## 2. Results

### 2.1. Effects of A009 on Cell Proliferation, Apoptosis, and Cell Cycle

**Figure 1:**
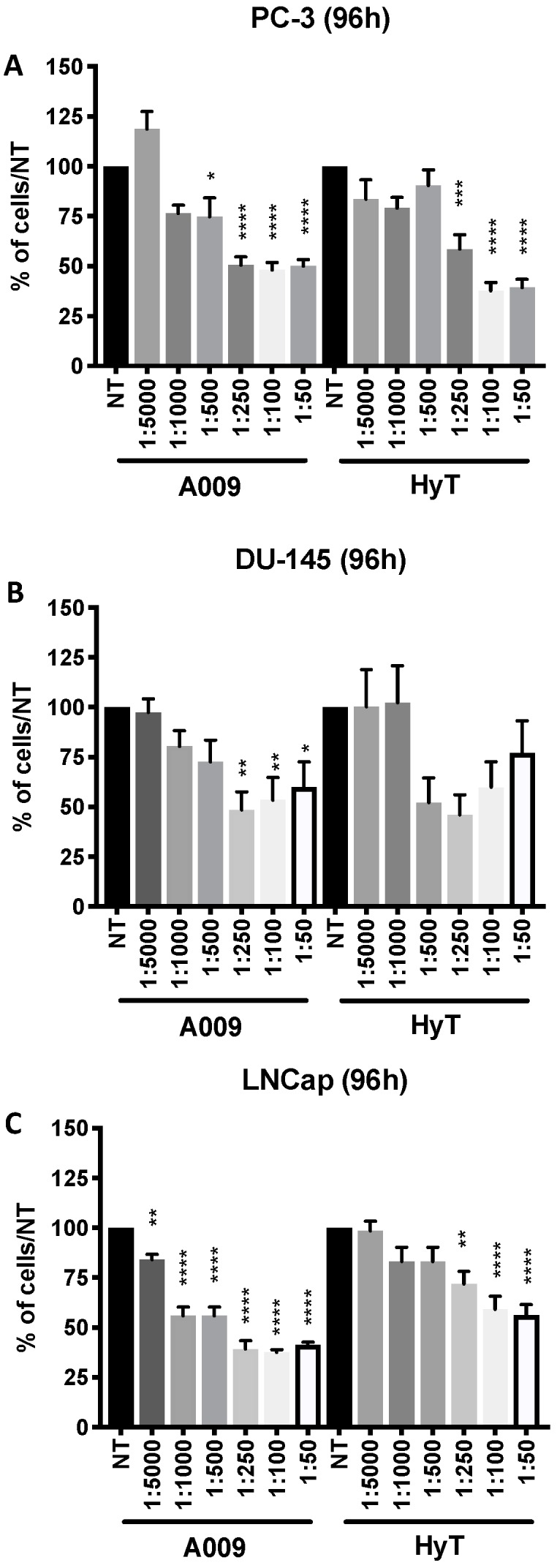
Effects of A009 on PC-3, DU-145, and LNCaP human prostate cancer cell proliferation. Graphs show the effects of A009 L4 and HyT, following 96 h of treatment on PC-3 (**A**), DU-145 (**B**), and LNCaP (**C**) PCa cell proliferation, as assessed by the MTT (3-(4,5-dimethylthiazol-2-yl)-2,5-diphenyltetrazolium bromide) assay. A009 L4 was more effective in inhibiting cell proliferation as compared with HyT, in a dilution-dependent manner. Not treated cells (NT) received complete RPMI medium. Results are shown as mean ± SEM.

To investigate the anti-proliferative effects of A009 we employed three PCa cell lines: hormone-sensitive (LNCaP) and human metastatic hormone-resistant PCa cells (PC-3, DU-145). Cells were treated with various dilutions (ranging from 1:50 to 1:5000) of A009 and compared with HyT alone, at the same concentrations as within the A009 extracts [18,19]. Two lots of A009 (L3 and L4) were used with similar results. Growth was detected at 48 and 96 h. MTT results (Figure 1 and Figure S1) showed that, starting from the 1:5000 dilution, A009 inhibited PC-3, DU-145, and LNCaP cell proliferation, and inhibition was significant at intermediate dilutions. A009 lot 4 (L4) was more effective in inhibiting cell proliferation, as detected at 96 h following treatment (Figure 1), as compared with HyT for LNCap cells, in a dilution-dependent manner, and similar for the other lines. We selected the A009 1:250 and 1:500 dilutions for further studies.

**Figure 2:**
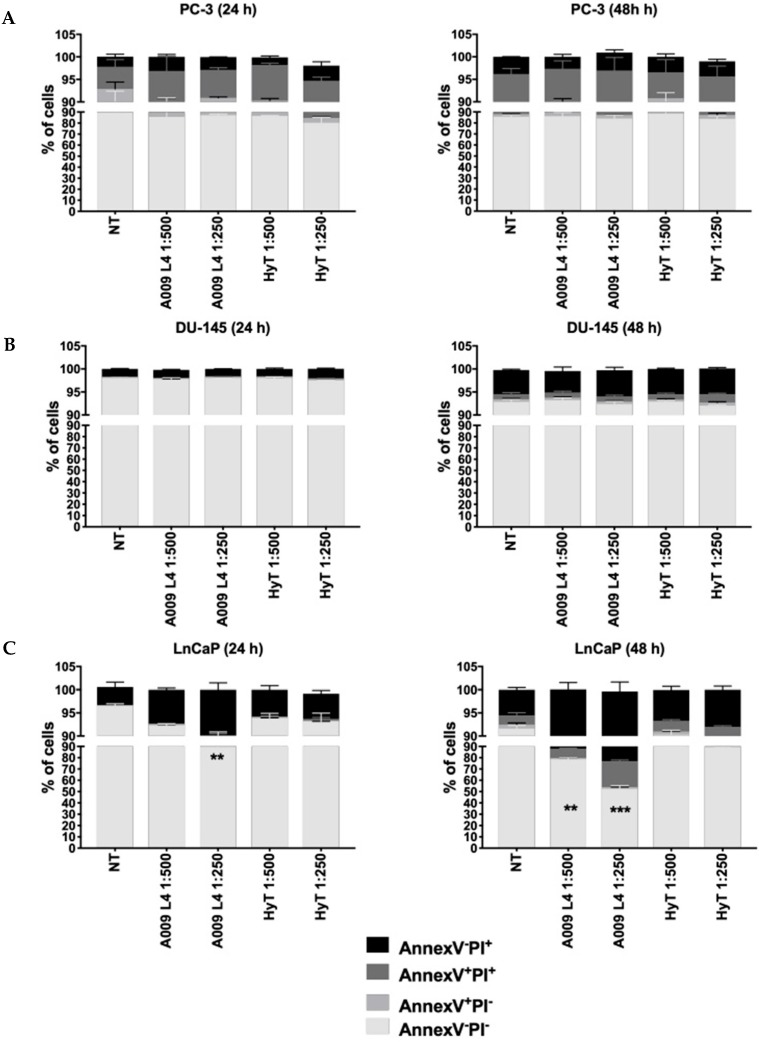
Effects of A009 (L4) on PC-3, DU-145 and LNCaP apoptosis. Apoptosis (24 and 48 h) was assessed by flow cytometry on PC-3 (**A**), DU-145 (**B**), and LNCaP (**C**) human prostate cancer cells. Induction of specific apoptosis phases was detected by gating annexin V^−^/PI^−^ cells (viable), annexin V^+^/PI^−^ cells (early apoptotic), annexin V^+^/PI^+^ cells (late apoptotic) and annexin V^−^/PI^+^ cells (dead). EtOH diluted (1:500 and 1:250) in complete RPMI was used as control vehicle for HyT, with no effects on induction of apoptosis (data not shown). Results are shown as mean ± SEM, ANOVA, ** *p* < 0.01, *** *p* < 0.001.

We observed moderate induction of apoptosis by A009 on PC-3 and DU-145 cells (Figure 2A,B) while LNCaP cells (Figure 2C) exposed to A009 exhibit significant induction of apoptosis at 24 h (A009 L4, 1:250) and at 48 h (A009 L4, 1:500 and 1:250), as compared to HyT at the same dilutions. Ethanol (EtOH) diluted (control vehicle for HyT, range of 1:500 to 1:250) in complete RPMI was used as control vehicle for HyT, with no effects on cell proliferation [19].

We also investigated the effects of A009 on cell cycle, in DU-145 and LNCaP PCa cell lines, following 24 and 48 h of treatment. We observed a trend of decreased ability to undergo the S-phase in DU-145 and LNCaP cells treated with A009, and in LNCaP cells at 24 and 48 h following treatment however, no statistical significance was found (Figures S1 and S2). EtOH diluted (1:500 or 1:250) in complete RPMI was used as control vehicle for HyT, with no effects on cell cycle [19].

### 2.2. Effects of A009 on Prostate Cancer Cell Adhesion

**Figure 3:**
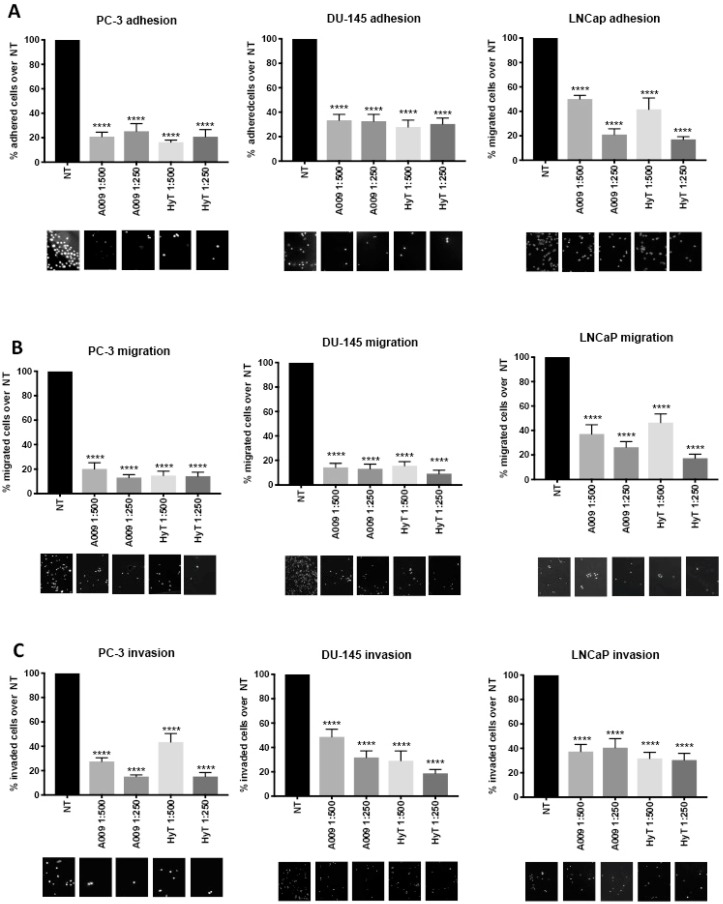
Effects of A009 on DU-145 and LNCaP human prostate cancer cell adhesion, migration, and invasion. PC-3, DU-145, and LNCaP were pre-treated for 24 h with A009 L4 or HyT, and their ability to prevent cell adhesion on fibronectin (**A**) migration on fibronectin (**B**) and invasion towards matrigel (**C**) was analyzed using Boyden chambers. Both A009 (dilution 1:500 or 1:500) and HyT were able to significantly inhibit cell adhesion, migration, and invasion in the three prostate cancer (PCa) cell lines. Representative images show adherent, migrated, and invading DU-145 and LNCaP cells at magnification 10×. Results are showed as mean ± SEM, ANOVA, **** *p* < 0.001.

We investigated the adhesion capabilities of PC-3, DU-145, and LNCaP human PCa cells, which were pre-treated for 24 h with A009 or HyT (1:500, 1:250). A009 significantly prevented PC-3, DU-145, and LNCaP adhesion (Figure 3A). A comparable effect was observed when cells were treated with HyT (Figure 3A). EtOH diluted (1:500 or 1:250) in complete RPMI was used as control vehicle for HyT, with no effects on cell adhesion [19].

### 2.3. Effects of A009 on Migration and Invasion in Human Prostate Cancer Cells

Migration and invasiveness of tumor cells are crucial steps for the development of malignancies and cancer progression [36,37]. The properties of A009 to prevent cell migration and invasion were investigated following pre-treatment for 24 h of PC-3, DU-145, and LNCaP cells in the Boyden chamber assay as described in the Materials and Methods section. Both A009 and HyT significantly inhibited migration (Figure 3B) and invasion though a reconstituted basement membrane (Figure 3C) of PC-3, DU-145, and LNCaP cells. EtOH diluted (1:500 or 1:250) in complete RPMI was used as control vehicle for HyT, with no effects on cell migration and invasion [19].

### 2.4. Effects of A009 on Pro-Angiogenic Factor Release in Human Prostate Cancer Cells

**Figure 4:**
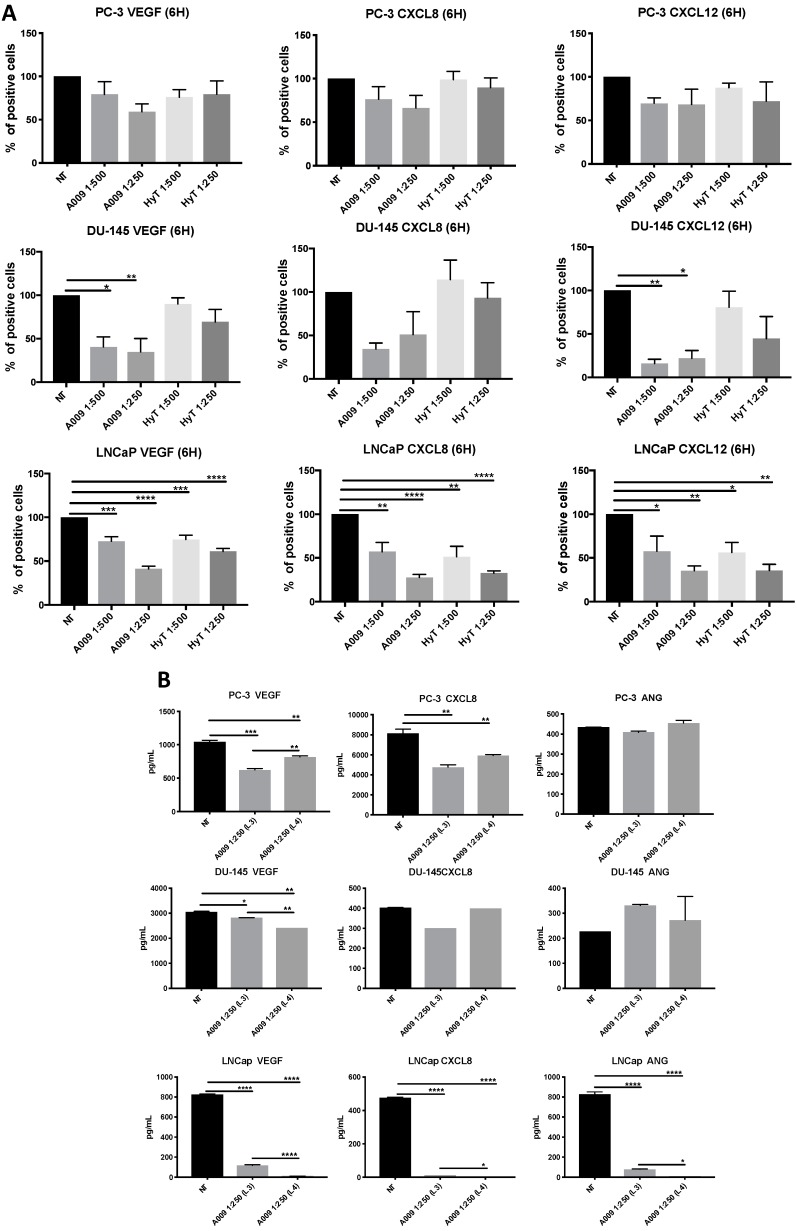
Cytokine profiling on PC-3, DU-145, and LNCaP human prostate cancer cells treated with A009. (**A**) PC-3, DU-145, and LNCaP were treated with A009 or HyT (dilution 1:500 and 1:250) for 6 h, and analyzed for cytokine production by flow cytometry. FACS analysis showed that A009 reduced VEGF, CXCL8, and CXCL12 release on PC-3, DU-145, and LNCaP PCa cell lines. (**B**) Secretome profiling on DU-145 and LNCaP secreted products, following 24 h treatment with A009 (L3 or L4, dilution 1:250), by BIOPLEX, showed the ability of A009 to inhibit production of VEGF and CXCL8 release in the three PCa cell lines investigated. The was particular strong in LNCap cells. Results are showed as mean ± SEM, ANOVA, * *p* < 0.05, ** *p* < 0.01, *** *p* < 0.001, **** *p* < 0.0001.

We investigated whether the A009 extracts were effective in limiting the release of pro-angiogenic factors in PC-3, DU-145, and LnCaP PCa cell lines. FACS analysis showed decreased production of VEGF, CXCL8, and CXCL12 by the three PCa cell lines, exposed to A009 at 1:500 and 1:250 dilutions, following 6 h of treatment (Figure 4A,B). HyT inhibitory effects were lower in the three PCa cell lines as compared to those exposed to the same dilutions of A009.

We validated our FACS data on VEGF and CXCL8, by evaluating the release of the same cytokines by BIOPLEX assay, described in the Materials and Methods section. Secreted products were collected from PC-3, DU-145, and LNCaP cells, following 24 h exposure to two different lots of A009 (L3 and L4), at 1:250 dilution. We observed a statistically significant reduction in VEGF and CXCL8 release by the three PCa cell lines exposed to the A009, confirming the FACS data. Angiogenin (ANG) was modulated only in LNCaP cells, following A009 treatment (Figure 4C).

### 2.5. A009 Inhibits NF-κB Signaling Pathway in Human Prostate Cancer Cells

**Figure 5:**
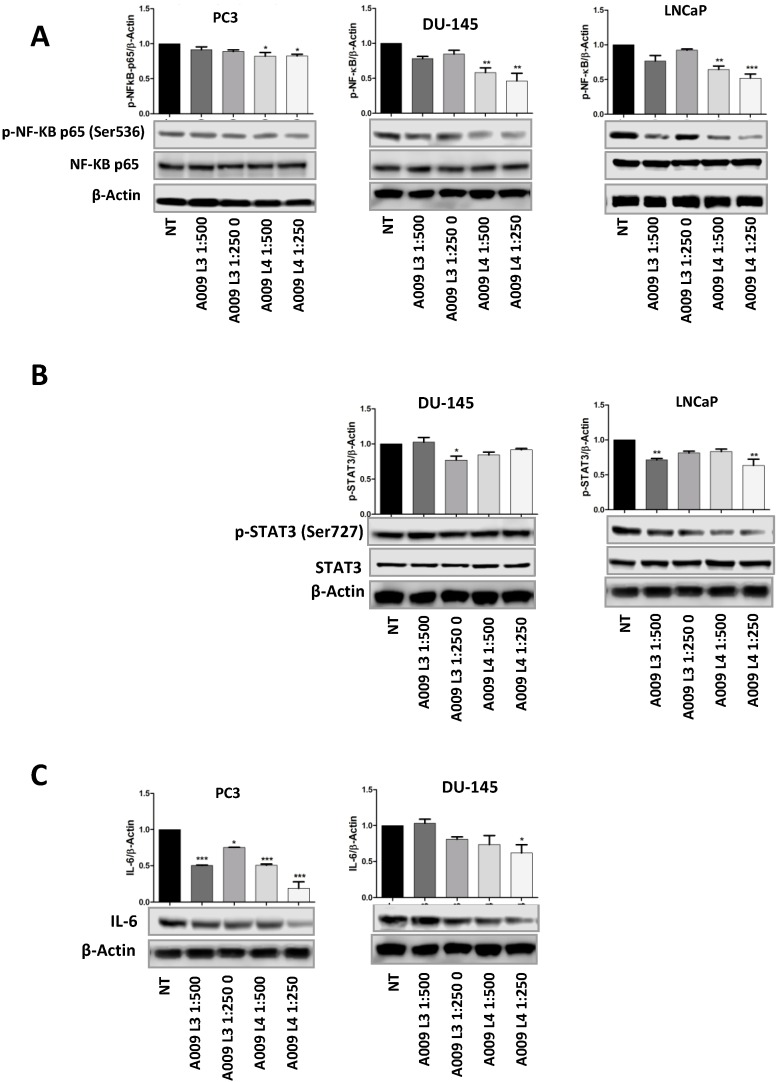
Effects of A009 on NF-κB, IL-6, and STAT3 production and activation in human prostate cancer cells. PC-3, DU-145, and LNCaP cells were treated for 24 h with A009 (L3 and L4, dilution 1:250, 1:500). (**A**) Effects of A009 (L3 and L4, dilution 1:250, 1:500) on phosphorylation of NF-κB (Ser536) were determined by Western blot. A009 was able to significantly lower activation of NF-κB in PCa cell lines. (**B**) A009 affects activation of p-STAT3 (Ser727) on DU-145 and LNCaP, and (**C**) downregulates IL-6 in PC-3 and DU-145 when treated with A009. Results are showed as mean ± SEM, ANOVA, * *p* < 0.05, ** *p* < 0.01.

A substantial body of evidence has confirmed that cancer-associated inflammation through the presence of inflammatory cells and the release of cytokines, chemokines, reactive oxygen species (ROS), and prostaglandins, favor PCa initiation and progression [38,39,40,41,42]. Given the antioxidant and anti-inflammatory properties, we explored the ability of A009 to target NF-κB on PCa cells. We found that PC-3, DU-145, and LNCaP PCa cell lines all exhibited reduction of NF-κB activity, following treatment with A009 for 24 h, normalizing to both β-actin (Figure 5A) and NF-κB total protein.

### 2.6. A009 Inhibits IL-6/STAT3 Signaling Pathway

It has been shown that NF-κB controls the expression of numerous genes, including that of interleukin 6 (IL-6) [43,44,45,46]. STAT3 is a point of convergence for numerous oncogenic signaling pathways and increased nuclear translocation and phosphorylation of STAT3 are related to prostate cancer growth, invasion, and metastasis [47,48]. IL-6/STAT3 signaling is crucial for maintenance of the stem cell phenotype [49] and considered to be a therapeutic target to treat advanced PCa [50,51,52]. We therefore evaluated the ability of A009 to interfere with IL6/STAT3 activation and phosphorylation in DU-145 and LNCaP PCa cells. We found significantly reduced phosphorylation of STAT3 (Ser727) at the protein level following 24 h of treatment (Figure 5B). A009 significantly reduced IL-6 at protein level (Figure 5C). LNCaP cells make very low levels of IL-6 [47,48,49], thus, its modulation was not investigated.

## 3. Discussion

Prostate cancer represents the second most common cancer in men and the fifth leading cause of cancer-related deaths worldwide [20,53]. The development of PCa is complex and has been reported to be determined by a combination of genetic, hormonal, and environmental factors [20,53]. Diet is one of the most modifiable risk factors for PCa [54,55]. Traditional knowledge and scientific reports demonstrate that, among nutraceuticals, olive oil is a source of biologically active compounds that can be used for the prevention of various diseases, including some type of cancers [14,56,57,58]. The production of olive oil implies the generation of important amounts of byproducts, in particular, olive mill wastewater (OMWW). Although a discarded material, OMWWs is extremely attractive for the presence of soluble polyphenols [57]. OMWW represents a problem for the olive oil production industry, being a polluting effluent able to modify soil and water quality, and negatively impacts the ecosystem [57]. We previously reported that OMWW contains high amounts of HyT and other polyphenols [18,19]. HyT, by preventing initiation and promotion/progression phases of carcinogenesis, has been shown to act as a chemopreventive agent in several cancer models [56,58,59,60,61]. We have shown that a polyphenol-rich OMWW extract, A009, interferes with the angiogenic process in endothelial cells in vitro and in vivo [18,19]. Moreover, we demonstrated that A009 prevents human colon cancer proliferation in vitro and tumor growth in vivo [19]. A009 extract represents a rich source of HyT from a waste product, without some highly caloric oil components. These oily fraction components might promote cancer [62,63].

**Figure 6:**
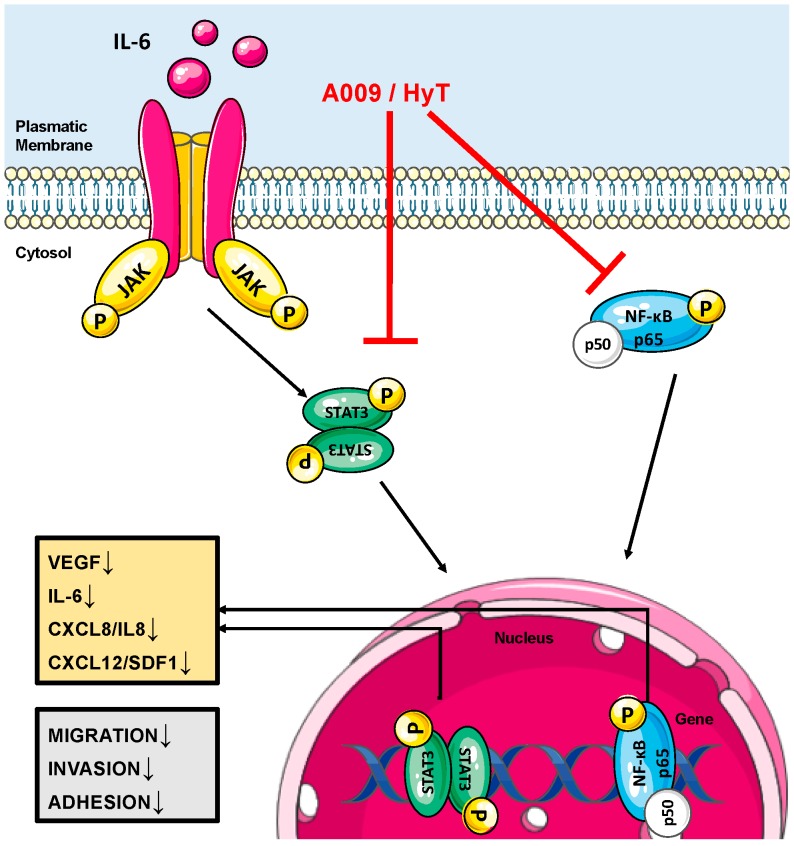
Schematic representation for the potential chemopreventive activities of A009 on prostate cancer cell lines in vitro. The cartoon summarizes the major findings at cellular and molecular levels associated with the targets of A009 identified in the manuscript, down arrow (↓) means downregulation of the protein or function.

A growing amount of data show that polyphenols can prevent prostate cancer development and invasiveness [64]. HyT has been reported to inhibit human prostate cancer (LNCaP and PC-3) cell proliferation and induce apoptosis [56,65,66]. Additionally, polyphenols are able to modulate signaling cascades involved in the growth and proliferation of tumor cells and in inflammation (e.g., MAPK, PI3K, and NF-κB) [7,10,67]. Here, we investigated the effects of OMWW-derived extract, A009, particularly rich in HyT, but also containing several preventive polyphenols, on human prostate cancer cells (Figure 6). We also tested two different lots, both with a similar composition [18,19]. Our results showed that that A009 prevented PC-3, DU-145, and LNCaP human prostate cancer cell proliferation, in vitro, in a time- and concentration-dependent manner. A009 was able to induce apoptosis in a statistically significant manner only in LnCaP cells, as for HyT, with limited toxic effects that were observed only in LNCaP PCa cells [66]. We also observed a trend on inhibition of the S-phase in treated cells, although not statistically significant. This is in keeping with the inhibition of the S-phase reported by Zubair et al., which used 2–6 times more concentrated HyT [66]. We identified 1:500 and 1:250 A009 dilutions as the most suitable and we further tested them in functional assays in vitro. We explored whether A009 could limit crucial events for tumor progression, such as adhesion, migration, and invasion, on in vitro models of human prostate cancer. Pre-treatment of PC-3, DU-145, and LNCaP cells with A009 for 24 h resulted in reduced cell adhesion, migration, and invasion of the two PCa cell lines in vitro.

Tumor cells need to interact with stromal components and recruit inflammatory and endothelial cells to fuel nutrients and disseminate to distant organs. This requires the secretion of pro-inflammatory and pro-angiogenic factors acting both at tissue and systemic levels [68,69,70]. We found that our A009 extracts limit, in vitro, the production and release of VEGF and CXCL8, as revealed by FACS (production of cytokines by the cells) and BIOPLEX analysis (secretion of cytokines), and CXCL12, suggesting that potential in vitro chemopreventive activities of A009 act by targeting soluble effectors mediating inflammatory angiogenesis (Figure 6).

We performed further biochemical analysis to identify molecular mechanisms associated with the functional properties exhibited by our A009 extracts.

The master transcription factor, NF-κB, has been recognized as the major effector of pro-inflammatory processes involved in PCa pathogenesis [30,31,32,33,34]. NF-κB has been correlated with cellular transformation, prostate cancer growth, lymph node metastases, resistance to chemotherapy, and disease outcome [30,32,34]. The NF-κB family of transcription factors have been shown to be important and implicated in prostate cancer [32,34,71,72]. Due to the many links between inflammatory signals and the malignant development of prostate cancer, targeting NF-κB has been suggested as a promising therapeutic option [73]. We found that our A009 extracts were able to reduce, in vitro, NF-κB activation in the PCa cell lines following 24 h of treatment (Figure 6).

The role of IL-6 in PCa progression has been widely reported and suggested as a candidate for targeted therapy of PCa. In addition to its role as an immunomodulatory cytokine, IL-6 acts as an autocrine and paracrine growth factor [74] and has been reported to be involved in prostate cancer cell growth by activating signaling pathways/signal transducers and activators of transcription factors, including the STAT family [75,76,77]. IL-6 has been suggested to activate the androgen receptor of PCa [78,79] in the regulation of vascular endothelial growth factor (VEGF) expression [80] and epithelial/mesenchymal transition, and metastasis of PCa [51]. A series of studies has reported elevated serum levels of IL-6 in androgen-independent prostate cancer, that is strongly correlated with development and progression of castration-resistant prostate cancer (CRPC) [77,81]. IL-6 is largely expressed in androgen-independent prostate cancer cell lines, and overexpression of IL-6 in LNCaP can lead to these cells being resistant to androgen deprivation therapy [75].

STAT3 is considered, along with NF-κB, to be a master regulator for tumor progression, acting by supporting inflammation and angiogenesis [43,44,47,48,49,50,51,52]. STAT3 and IL-6 have a feed-forward loop, each one regulating the other [82]. Given the key role of IL-6/STAT3 on PCa progression, we also evaluated the in vitro effect of A009 extracts on this axis. Following 24 h of treatment, A009 was able to reduce IL-6 and STAT3 phosphorylation (Ser727) in DU-145, showing a potential cancer-preventive role by blocking IL-6/STAT3 signaling in vitro. In addition, the properties of A009 to inhibit STAT3 phosphorylation and activity was also confirmed in LNCaP PCa cells. Our results support the idea of repositioning a food waste-derived material for nutraceutical employment, with environmental and industrial cost management benefits. The highest modulations were detected on hormone dependent LNCap cells, a further hint that OMWW containing products could be useful in PCa prevention and interception.

## 4. Materials and Methods

### 4.1. Reagents and Chemicals

The synthetic hydroxytyrosol (HyT), ≥98% in purity, was purchased from Cayman Chemicals (Ann Arbor, MI, USA). HyT was dissolved in ethanol (EtOH) and added to the medium, while EtOH vehicle alone was also used as a control. Two different lots of A009 (L3 and L4) were used. When not specified, experiments showing only one A009 extract were performed using the L4 lot.

### 4.2. Preparation of A009 and Phenolic Quantification

OMWWs were kindly provided by Agriturismo La Vialla (Castiglion Fibocchi, Arezzo, Italy) and used to obtain the phenol-rich purified extract A009 (Patents No. 1420804; No. 1420805). The experiments were performed using two different batches of A009 (batch L3 and L4, Table S1). A009 was obtained from OMWW using two sequential cross-flow filtration processes, as previously described [18]. Briefly, microfiltration (MF) was performed on a pilot plant equipped with tubular ceramic module membranes in alumina oxide with a MWCO of 0.45 microns. The MF permeate was further concentrated by reverse osmosis (RO) in a polyamide spiral wound module (Microdyn Nadir, Wiesbaden, Germany) with a filtering surface of 7 m^2^. The RO permeate, representing ultrapure water, was discarded. Finally, the RO concentrate, with a volume concentration ratio (VCR) of 3.6, constituted the A009. Phenolic composition of A009 was determined using high-performance liquid chromatography (HPLC) analysis. Quantification revealed that the major phenolic component of A009 is HyT, along with several other phenolic compounds (Table S1).

### 4.3. Cell Cultures

All experiments were performed using three cell lines of prostate cancer, human metastatic hormone-sensitive LNCaP and hormone-resistant cells PC-3, and DU-145. All prostate cancer cell lines were purchased from ATCC and grown in RPMI 1640 medium (Euroclone, Pero, MI, Italy) supplemented with 10% fetal bovine serum (FBS, Gibco, Thermo Fisher Scientific, Rodano, MI, Italy), 2 mM l-glutamine (Sigma-Aldrich, Saint Luis, MO, USA), 100 U/mL penicillin, and 100 µg/mL (Sigma-Aldrich, Saint Luis, MO, USA). LNCaP was grown on culture dishes coated with 0.01% polylysine solution (Sigma-Aldrich). All cell lines were maintained at 37 °C in 5% CO_2_.

### 4.4. In Vitro Cell Proliferation Assay

Cell proliferation was assessed by MTT (3-(4,5-dimethylthiazol-2-yl)-2,5-diphenyltetrazolium bromide; Sigma Aldrich, Milan, Italy). PC-3, DU-145, and LNCaP cancer cells (10^3^ cells/well) were seeded into a 96-well plate. Following cell adhesion, fresh complete medium with decreasing dilutions (ranging from 1:50 to 1:10,000) of A009 or HyT were added for 24 to 96 h. EtOH dissolved in the RPMI complete medium was used as control vehicle. MTT reagent 5 (mg/mL) was added to cell cultures, which was then replaced with 100 μL of DMSO, and the amount of solubilized formazan was quantified at 570 nm in a SpectraMax M2 (Molecular Devices, Sunnyvale, CA, USA).

### 4.5. Detection of Apoptosis In Vitro

PCa cells were treated with two dilutions of A009 (1:250; 1:500) and HyT with the same concentrations for 24 and 48 h. Control cells receive complete medium alone. Apoptosis was detected by PI (Sigma Aldrich) and anti-annexin V-FITC (Becton Dickinson-BD, Franklin Lakes, NJ, USA) staining, followed by flow cytometry analysis, using a BD FACSCantoII flow cytometer. Flow data were analyzed with the FACSDiva 6.1.2 software (Becton Dickinson-BD, Franklin Lakes, NJ, USA).

### 4.6. Detection of Cell Cycle Arrest In Vitro

PC-3, DU-145, and LNCaP cells were treated with two concentrations of A009 (1:250; 1:500) and HyT with the same concentrations as in the two batches of A009, for 24 and 48 h. Control cells received complete medium alone (negative control) or vincristine (10 µM, positive control). At each time point, cells were detached and fixed in cold 70% ethanol. DNA was stained with 100 μg/mL DAPI 2 µg/mL (Sigma-Aldrich) in 1× PBS solution. Stained nuclei were analyzed for V450 fluorescence signal using a FACSCanto flow cytometer. G0/G1, S, G2/M, and apoptotic phase of the cell cycle were determined using the FACSDiva 6.1.2 (Becton Dickinson-BD, Franklin Lakes, NJ, USA) and FlowLogic software (Miltenyi Biotec, Bergisch Gladbach, Germany).

### 4.7. Cell Adhesion Assay In Vitro

Cell adhesion assay was performed as previously described [83]. PC-3, DU-145, and LNCaP cells were treated for 24 h with A009 (1:250; 1:500) and HyT (1:250; 1:500). Positive controls received complete medium alone or FBS (10%). Following treatment, 3 × 10^3^ cells were seeded on a 4-well chamber slide coated with fibronectin (2 µg/mL). Following 90 min of incubation, cells were washed, fixed with 4% paraformaldehyde (PFA), and then stained with DAPI (Sigma Aldrich). Assays were performed in triplicates. Cells within 5 blind fields per well were randomly counted to determine the number of adherent cells resulting from each treatment, using a Zeiss Microscope associated with a Nikon camera. Experiments were performed in triplicate, three times.

### 4.8. Migration and Invasion Assays In Vitro

Migration and invasion assays were performed using a modified Boyden chamber, as previously described [36,84]. PC-3, DU-145, and LNCaP cells (5 × 10^4^), treated with A009 and HyT for 24 h, were washed with PBS, resuspended in serum-free RPMI medium, and placed in the upper compartment of the Boyden chamber. RPMI complete medium was added in the lower compartment to induce migration and invasion, and in the related in vitro assays. Polycarbonate filters, of 10 µm pore size, pre-coated with matrigel (1 mg/mL, Becton Dickinson-BD, Franklin Lakes, NJ, USA) for the chemoinvasion assay or with collagen IV (50 µg/mL, Sigma Aldrich) for the chemotaxis assay, were used as the interface between two Boyden chamber compartments. Following 6 h (chemotaxis) or 24 h (chemoinvasion) of incubation at 37 °C in 5% CO_2_, the filters were recovered, and cells on the upper surface were mechanically removed with a cotton swab and migrated or invaded cells on the lower filter surface were fixed with absolute ethanol and stained with DAPI. Migrated/invaded cells for each treatment were counted in a double-blind manner in 5 random fields using a Zeiss Microscope associated with a Nikon camera (Axio Observer A1, Zeiss, Germany). Experiments were performed in triplicate.

### 4.9. Western Blotting Analysis

Cells were lysed in RIPA buffer containing protease and phosphatase inhibitor cocktails (Roche Diagnostics GmbH). Proteins (30 µg) were loaded on NuPAGE Novex 4%–12% Bis-Tris Gel (Life Technologies) and transferred to a PVDF membrane Amersham Hybond (GE Healthcare Bio-Sciences, Pittsburgh, PA, USA). Membranes were incubated overnight at 4 °C with the primary antibodies: anti-NF-κB p65, anti-p-STAT3, anti-IL-6 (all from Cell Signaling Technology). The secondary antibody peroxidase-linked anti-rabbit IgG or anti-mouse IgG (GE Healthcare Bio-Sciences, Pittsburgh, PA, USA) was diluted at 1:3000 for 1 h at room temperature. Specific protein bands were detected using Pierce ECL Western Blotting Substrate (ThermoFisher Scientific). Protein expressions were normalized to beta-actin at 1:5000 (Abcam). Western blot data were analyzed using ImageJ software to determine the optical density (OD) of the bands.

### 4.10. Analysis of Intracellular Cytokines In Vitro

The production of intracellular cytokines was performed by flow cytometry. PC-3, DU-145, and LNCaP PCa cells were treated for 6 h with 1:500 and 1:250 A009 or HyT, in complete RPMI medium supplemented with 10 ng/mL phorbol 12-myristate 13-acetate (PMA; Sigma-Aldrich, St. Louis, MO, USA), 500 ng/mL ionomycin (Sigma-Aldrich), and protein transport inhibitor brefeldin A (Golgi Plug) (Becton Dickinson-BD, Franklin Lakes, NJ, USA)). Following 6 h of stimulation, cells were harvested and treated with Cytofix/Cytoperm fixation and permeabilization kit (BD). Cells were stained with phycoerythrin (PE)-conjugated anti-human VEGF (R&D Systems), PE-conjugated anti-human CXCL-8 (R&D Systems), and PE-conjugated anti-human CXCL12 (BD). Negative controls included directly labeled isotype-matched irrelevant mAbs (BD). The expression of specific cytokines and angiogenic growth factors were determined by flow cytometry, using a BD FACSCantoII analyzer.

### 4.11. Cytokine Profiling on PCa Cells Secreted Products

Cytokine profiling was investigated by secretome analysis. Following 24 h of treatment of the PCa cells, supernatants were collected, centrifuged to remove residual dead cells and debris, and concentrated using Concentricon devices (Millipore, Temecula, CA, USA), with a 3 kDa membrane pore cut-off, to obtain concentrated supernatants. Cytokine release profiling was performed using the Bio-Plex Pro Assays (Bio-Rad Laboratories, Hercules, CA, USA) in a customized cytokine panel.

### 4.12. Statistical Analysis

The statistical significance between multiple datasets was determined by one-way ANOVA using GraphPad Prism 7 and 8. Data are expressed as means ± SEM or SD.

## 5. Conclusions

Our results demonstrate that purified extract A009 from the waste material OMWW exert promising preventive activities, in vitro, on human prostate cancer cells. We demonstrated that our A009 extracts target NF-κB and the IL-6/STAT3 axis in vitro, providing a rationale for the potential use of a water-soluble olive polyphenol-rich nutraceutical product in a potential chemopreventive approach.

## Supplementary Materials

Supplementary materials can be found at http://www.mdpi.com/1422-0067/20/2/307/s1.
