# Supplementary material for: Downregulation of Pro-Inflammatory and Pro-Angiogenic Pathways in Prostate Cancer Cells by a Polyphenol-Rich Extract from Olive Mill Wastewater
Source: Int J Mol Sci. 2019 Jan 14;20(2):307. doi: 10.3390/ijms20020307 (PMC6359159; doi:10.3390/ijms20020307)
Supplement: Supplementary file 1 [file ijms-20-00307-s001.pdf]

Suppl. Table 1

PHENOLIC COMPOUND	A009 BATCH 3 (g/L)	A009 BATCH 4 (g/L)
Hydroxytyrosol glucoside	1.69	1.91
Hydroxytyrosol	5.72	5.50
Tyrosol	ND	0.69
Chlorogenic acid	0.10	0.13
b-hydroxyverbascoside isomer 1	0.14	0.23
b-hydroxyverbascoside isomer 2	0.17	0.23
Verbascoside	1.32	1.07
Caffeoyl ester of secologanoside	0.20	0.23
Decarboxymethyloleuropein aglycon	0.28	0.16
Oleouropein aglycon	0.22	0.21
6'-p-coumaroyl secologanoside	0.40	0.35
Rutin	ND	ND
Luteolin-7-o-glucoside	ND	ND

Suppl. Figure 1

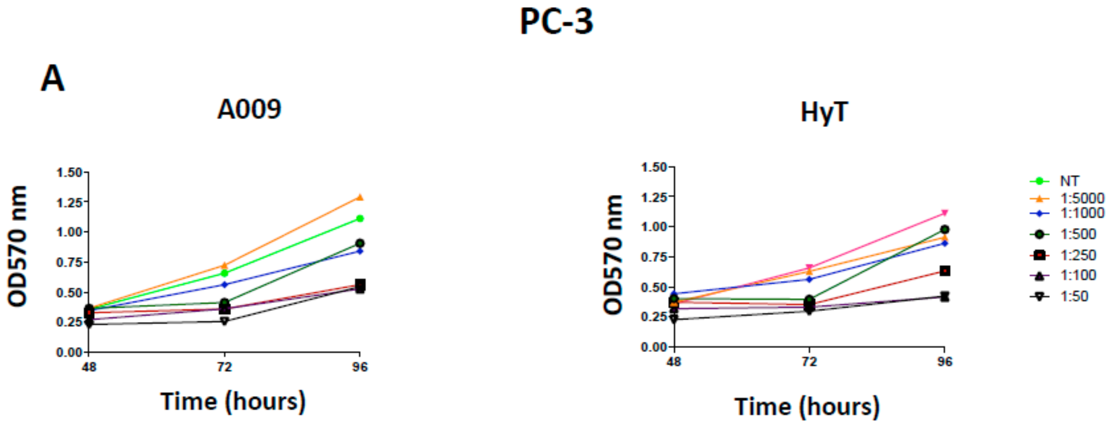

DU-145

B

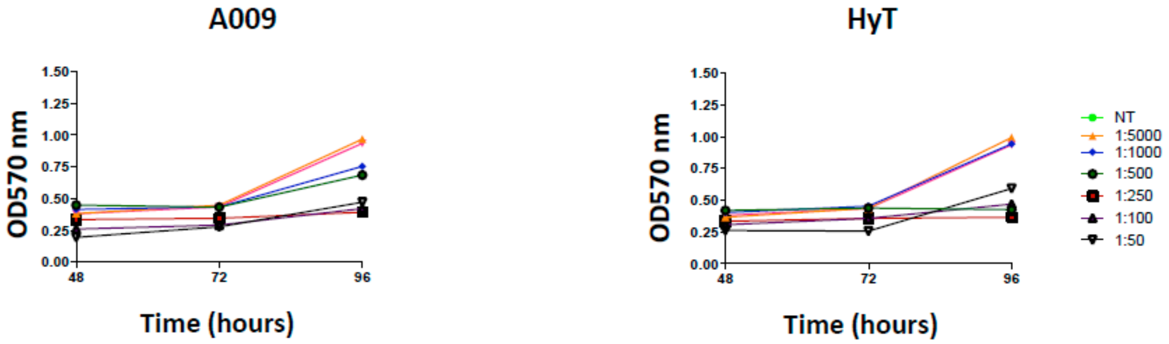

LNCaP

LNCaP

C

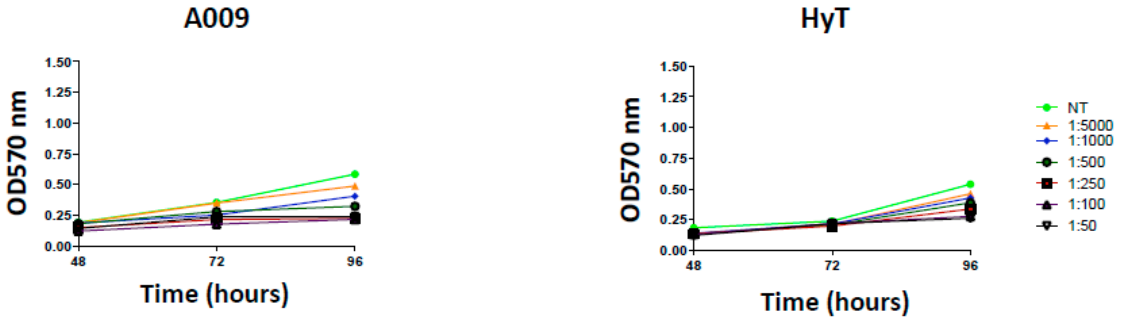

Suppl. Figure 2

A

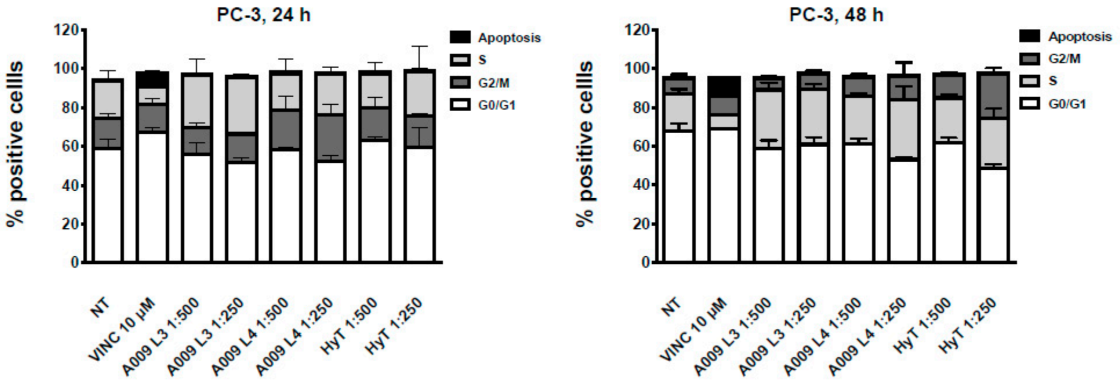

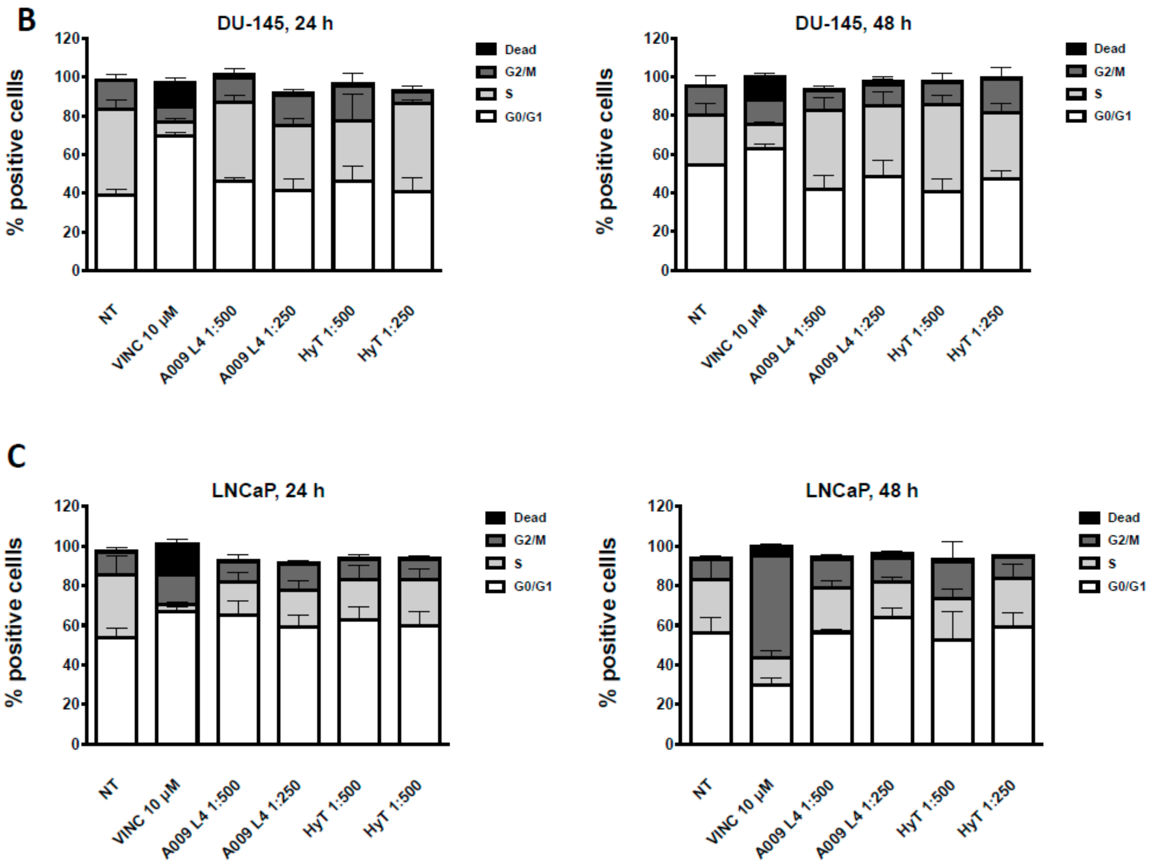
B
DU-145, 24 h
DU-145, 48 h
% positive celils
Dead
G2/M
S
G0/G1
NT
VINC 10 μM
A009 L4 1:500
A009 L4 1:250
HyT 1:500
HyT 1:250
C
LNCaP, 24 h
LNCaP, 48 h
120
100
80
60
40
20
0
